# Circadian Effects of Drug Responses

**DOI:** 10.1146/annurev-bioeng-082120-034725

**Published:** 2021-03-31

**Authors:** Yaakov Nahmias, Ioannis P. Androulakis

**Affiliations:** 1 Center for Bioengineering, School of Computer Science and Engineering, Hebrew University of Jerusalem, Jerusalem 91904, Israel; 2 Department of Biomedical Engineering and Department of Chemical & Biochemical Engineering, Rutgers University, Piscataway, New Jersey 08854, USA;; 3 Department of Surgery, Robert Wood Johnson Medical School, Rutgers University, Piscataway, New Jersey 08854, USA

**Keywords:** circadian, pharmacokinetics, pharmacodynamics, endocrine, chronopharmacology

## Abstract

Circadian rhythms describe physiological systems that repeat themselves with a cycle of approximately 24 h. Our understanding of the cellular and molecular origins of these oscillations has improved dramatically, allowing us to appreciate the significant role these oscillations play in maintaining physiological homeostasis. Circadian rhythms allow living organisms to predict and efficiently respond to a dynamically changing environment, set by repetitive day/night cycles. Since circadian rhythms underlie almost every aspect of human physiology, it is unsurprising that they also influence the response of a living organism to disease, stress, and therapeutics. Therefore, not only do the mechanisms that maintain health and disrupt homeostasis depend on our internal circadian clock, but also the way drugs are perceived and function depends on these physiological rhythms. We present a holistic view of the therapeutic process, discussing components such as disease state, pharmacokinetics, and pharmacodynamics, as well as adverse reactions that are critically affected by circadian rhythms. We outline challenges and opportunities in moving toward personalized medicine approaches that explore and capitalize on circadian rhythms for the benefit of the patient.

## THE RHYTHM OF LIFE

Homeostasis is a term introduced by physicians Walter Cannon (1) and Claude Bernard (2) to describe the myriad actions of physiological systems to maintain the relative constancy of the fluid environment surrounding cells. Such a fine equilibrium is the result of the coordinated actions of well-regulated active and passive physiological processes allowing an organism to respond and adapt to its changing environment (3). The concept of stress, introduced in early research by Hans Seyle (4), was eventually used to encompass all likely threats to homeostasis and the realization that numerous adaptive mechanisms are activated once the host detects external threats, often mediated via foreign substances that drive the stress response. The result of this process is the establishment of a new equilibrium in the body that considers the presence of these foreign substances. The breakdown of this finely tuned balance, often quantified through the deviation of levels of key biochemical entities and physiological processes from desired set points, is often associated with disease (5). Pharmacological interventions often aim to restore these biochemical entities to their expected homeostatic values. For example, statins were developed to reduce increased plasma cholesterol levels. Therefore, homeostasis (and, by extension, health) is often perceived as a relatively constant manifestation of biomarkers at appropriate levels, while deviation from these levels is considered a sign of an underlying disease.

**Figure 1:**
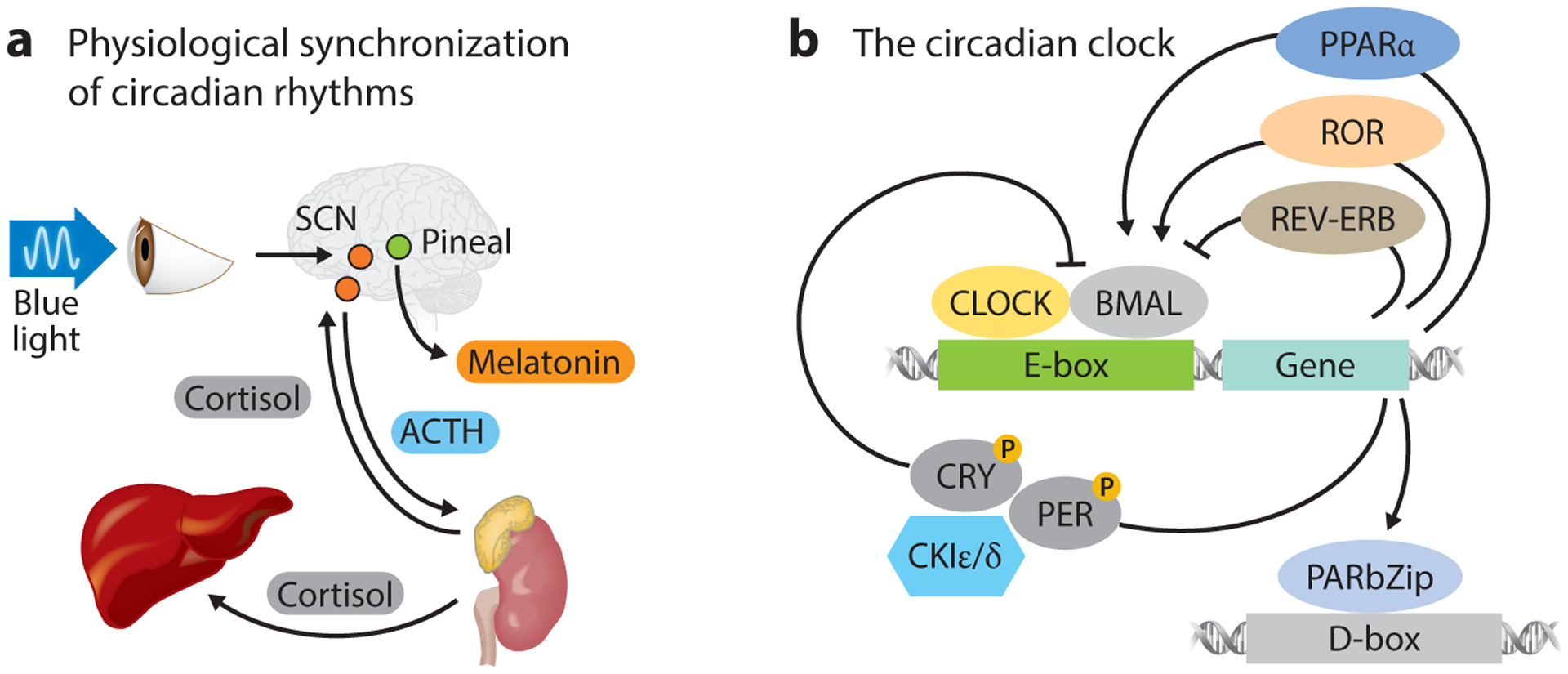
(*a*) Schematic of blue light–stimulated SCN control of hormone secretion in synchronization of the cell-autonomous clocks of peripheral tissues. Cortisol levels spike during early morning hours, while melatonin peaks at night. (*b*) Schematic of the canonical transcriptional feedback loop controlling cell-autonomous clocks. Cell-autonomous clocks are composed of a transcriptional feedback loop in which CLOCK and BMAL1 bind E-box elements on the promoters of PER and CRY that suppress the CLOCK/BMAL1 complex, inhibiting their own production. Abbreviations: ACTH, adrenocorticotropic hormone; PARbZip, proline and acidic amino acid–rich basic leucine zipper; PPAR, peroxisome proliferator–activated receptor; ROR, retinoic acid receptor–related orphan receptor; SCN, suprachiasmatic nucleus.

The ability to measure time and predict day/night cycles allows organisms across the biosphere to anticipate changing environmental conditions. These biological rhythms drive fluctuations in the homeostatic set points discussed above (6). For example, core temperature in primates changes by 1°C between day and night. Other rhythms include changes in activity, behavior, metabolism, reproductive capacity, sleep, and immune response (7–10). In mammals, this timing system, referred to as a circadian clock, exhibits a strict hierarchy wherein a central pacemaker located in the suprachiasmatic nucleus (SCN) continuously synchronizes a myriad of clocks in peripheral tissues using hormonal and nerve signaling (Figure 1a) (11). The molecular mechanism of the cell-autonomous clock was identified first in neurons of the SCN, and subsequently in most tissues, and exhibits autonomous, self-sustained oscillations driven by transcriptional and translational feedback loops (12). Briefly, the CLOCK and brain and muscle ARNT-like 1 (BMAL1) proteins form a heterocomplex and activate transcription of their own repressors, *Per1/2* and *Cry1/2* (13). CLOCK/BMAL1 heterodimers bind to the E-box region of *Per1/2* and *Cry1/2* genes and activate the transcription of these genes. Period (PER) and cryptochrome (CRY) proteins accumulate as a result of this transcriptional activation and form PER/CRY heterodimer complexes, which translocate to the nucleus (14). Autorepression of *Per* and *Cry* genes by their own products, PER and CRY, occurs through inhibition of CLOCK/BMAL1-induced transcription by the nuclear PER/CRY complex (13, 15–17). PER and CRY proteins also suppress the activation of REV-ERBα, which is promoted by the CLOCK/BMAL1 complex (18), while REV-ERBα negatively regulates transcription of BMAL1. Other transcription factors, such as peroxisome proliferator–activated receptor α (PPARα) and retinoic acid receptor–related orphan receptor (ROR), positively regulate the BMAL1 complex, allowing the mechanism to be influenced by hormonal or nutritional signals (Figure 1b).

Cell-autonomous clocks often oscillate at an intrinsic phase with periods a little different than 24 h and, thus, need to be synchronized within and across multiple tissues and organs. This task is undertaken by the SCN master clock, which is entrained by light/dark cycles and synchronizes cell-autonomous clocks throughout the body. Photosensitive ganglion cells within the retina that are exposed to short-wavelength or high-intensity light signal the SCN in the anterior part of the hypothalamus to transduce information to peripheral clocks both within and outside the central nervous system (19, 20). The SCN does so by, for instance, controlling the rhythms of the endocrine and autonomic nervous systems, which in turn communicate rhythmic signals to the periphery, acting as entrainers of the molecular clock in cells, tissues, and organs throughout the body (21–23). Recent genome-wide studies in rats (24) and mice (25) convincingly confirmed the existence of and tight coordination between these clocks across tissues and organs. These rhythmic changes in gene expression drive oscillations in a plethora of signaling, transport, metabolic, and physiologic processes that exhibit circadian rhythmicity. The temporal distribution of physiological activities in peripheral cells, tissues, and organs, coordinated and mediated by endocrine hormones and the autonomic nervous system, is centrally orchestrated by the rhythms set by the SCN. As a result, an intricate view of homeostasis was established—one that views a living host as a collection of finely tuned rhythmic processes controlling every aspect of physiology (26, 27).

Because life evolved in an environment characterized by predictable changes, we observe the establishment of anticipatory responses, driven by circadian rhythms, that lead to adjustment of physical activity, behavior, food intake, energy metabolism, immune function, and reproductive activity. These predictable changes, determined by the influence of day/night cycles due to Earth’s 24-h rotation around its axis and annual rotation around the Sun, establish a more predictive homeostasis. Accordingly, the term reactive homeostasis refers to unpredictable stressors that prompt an acute reaction (28). Predictive homeostasis is an extremely powerful concept leading to segregation of nocturnal (in rodents) and diurnal (in humans) activities during precise periods during the day, indicating the profound effect of temporally distributed physiology (28). The importance of the localization of activity in the proper period has been amply demonstrated in epidemiological studies (29). For example, night-shift workers are four times more likely to become diabetic and three times more likely to be obese than day-shift workers. Such active/inactive or light/dark cycles, and their associated physiological functions, are so precise and reproducible that they have resulted in the development of homeostatic mechanisms that have adopted this periodicity (30).

This nested cascade of rhythmic processes creates intriguing opportunities and formidable complications in the context of pharmacologic interventions. Drugs are multifactorial mediators, technically considered voluntary xenobiotics, that engage in a wide range of processes from the time they are delivered to the host (human or animal) until the time they exert their pharmacologic function at their corresponding target site. Every aspect of a drug’s administration [absorption, distribution, metabolism, excretion, pharmacodynamics (PD), and mechanism of action], as well as the disease mechanisms and symptomatology, is directly or indirectly enzyme mediated, either because the processes involved in the processing of the drug molecules are enzyme catalyzed or because the drug itself targets enzymes so as to increase or decrease their mediated actions. As such, it is not surprising that either the enzymes involved in the processing of a drug or the enzymes the drug targets are regulated by the circadian timing system. It has been estimated that more than 15% of the hepatic transcriptome and a significant fraction of the plasma metabolome oscillate (31, 32).

In the remainder of this review, we explore a number of these intricacies in exemplifying the challenges and opportunities in considering circadian rhythms in the context of pharmacology. Specifically, we address the following questions: (*a*) how circadian rhythms affect the PD and pharmacokinetics (PK) of the drug; (*b*) how circadian rhythms affect the mechanism of action of the drug and/or the mechanism of disease on which the drug acts; (*c*) how drugs affect the intrinsic circadian rhythms of the host; (*d*) how circadian rhythms become pharmacological targets; (*e*) how drug administration interferes with the host’s circadian rhythms; and finally (*f*) whether drug administration can be optimized and, if so, what should be optimized (see the sidebar titled Transcriptional Control of Metabolism).

## RHYTHMS OF HOMEOSTASIS AND THERAPEUTICS

**Figure 2:**
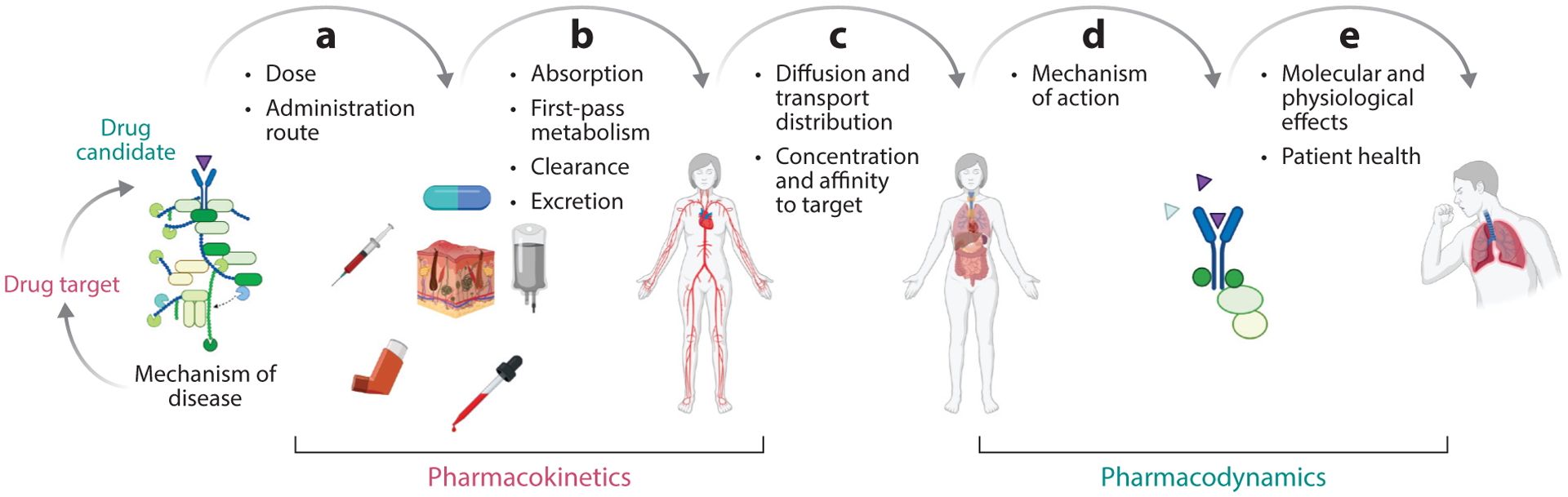
Schematic of the therapeutic process, (*a*) assuming that a drug and its relation to the mechanism of the disease have been identified and that an appropriate decision regarding dosing and administration route has been made. (*b*) This sequence comprises the distribution of the drug, which is determined on the basis of its absorption, first-pass metabolism, clearance, and excretion. (*c*) Once the drug enters the systemic circulation, the active ingredient is transported to the target site, hopefully at an appropriate concentration and with the desired selectivity and affinity to the target. (*d*) At this point, the mechanism of action of the drug is set in motion, driving the regulation of relevant and critical components of the disease mechanism and thereby leading to (*e*) molecular and physiological responses that ameliorate patient health. Figure adapted from images created with BioRender.com.

The therapeutic process often involves several steps, starting with drug delivery, specifically its dose and route of administration. The drug dose is often correlated with the amount of drug delivered to its intended target. Administration route is a more varied and complex parameter, ranging from oral administration to intravenous infusion, inhalation, or even ocular injection. Furthermore, the frequency and timing of delivery play an important role in the process. Once in the body, the active ingredient needs to be released, absorbed, metabolized, cleared, and excreted. The combination of these steps defines the drug PK, which describes what the body does to the drug. PK encompasses the transportation and distribution of the active molecule along its route to the drug target at concentration levels that are effective but not toxic, in order to engage a specific mechanism of action that can then induce or suppress a specific physiological set point and restore homeostasis. PD, the second part of the therapeutic process, describes what the drug does to the body (Figure 2).

**TRANSCRIPTIONAL CONTROL OF METABOLISM**

Nuclear receptors are a large family of ligand-activated transcription factors that play an important role in hormone control and homeostasis while responding to and influencing the circadian clock. They represent the largest family of transcription factors in humans, consisting of 48 different members that directly sense and respond to hormones, metabolites, vitamins, and xenobiotics (33). Hormone receptors, such as the glucocorticoid and thyroid hormone receptors, respond directly to changing concentrations of cortisol and thyroid hormones throughout the day. In addition, direct activation by metabolites, such as glucose, bile, or fatty acids, allows cells to rapidly react to metabolic changes by closing negative feedback loops (34). For example, glucose absorbed during the active period activates liver X receptor α (LXRα), which induces fatty acid synthesis, depleting glucose from circulation. Hours later, fatty acids released during fasting activate PPARα, blocking LXRα and inducing lipid peroxidation, thereby priming the body for rest. Another regulatory loop involving farnesoid X receptor (FXR), small heterodimer partner (SHP), and liver receptor homolog 1 leads to regulation of bile acid synthesis, uptake, and secretion. Interestingly, 25 of 45 nuclear receptors expressed in mice were found to rhythmically cycle (35). These include nuclear receptors directly regulated by the CLOCK/BMAL1 dimer, such as REV-ERBα and PPARα, as well as the constitutive androstane receptor, glucocorticoid and thyroid hormone receptors, FXR, and SHP, which play a critical role in hormone response, drug metabolism, and bile acid production (35).

Recent genome-wide transcriptomic studies across multiple tissues in mice (25), rats (24), primates (36), and especially humans (37, 38) have revealed that a substantial fraction of the genome exhibits strong, clear, 24-h circadian oscillations. This finding implies that a significant proportion of genes, and by extension their gene products and most likely their regulated functions, exhibit strong time-of-day dependence and are regulated, directly or directly, by clock genes (39, 40). Careful studies of major metabolic organs involved in drug metabolism, such as the liver, emphasize this temporal dependence at the proteomic (41) and metabolomic levels (42).

**Figure 3:**
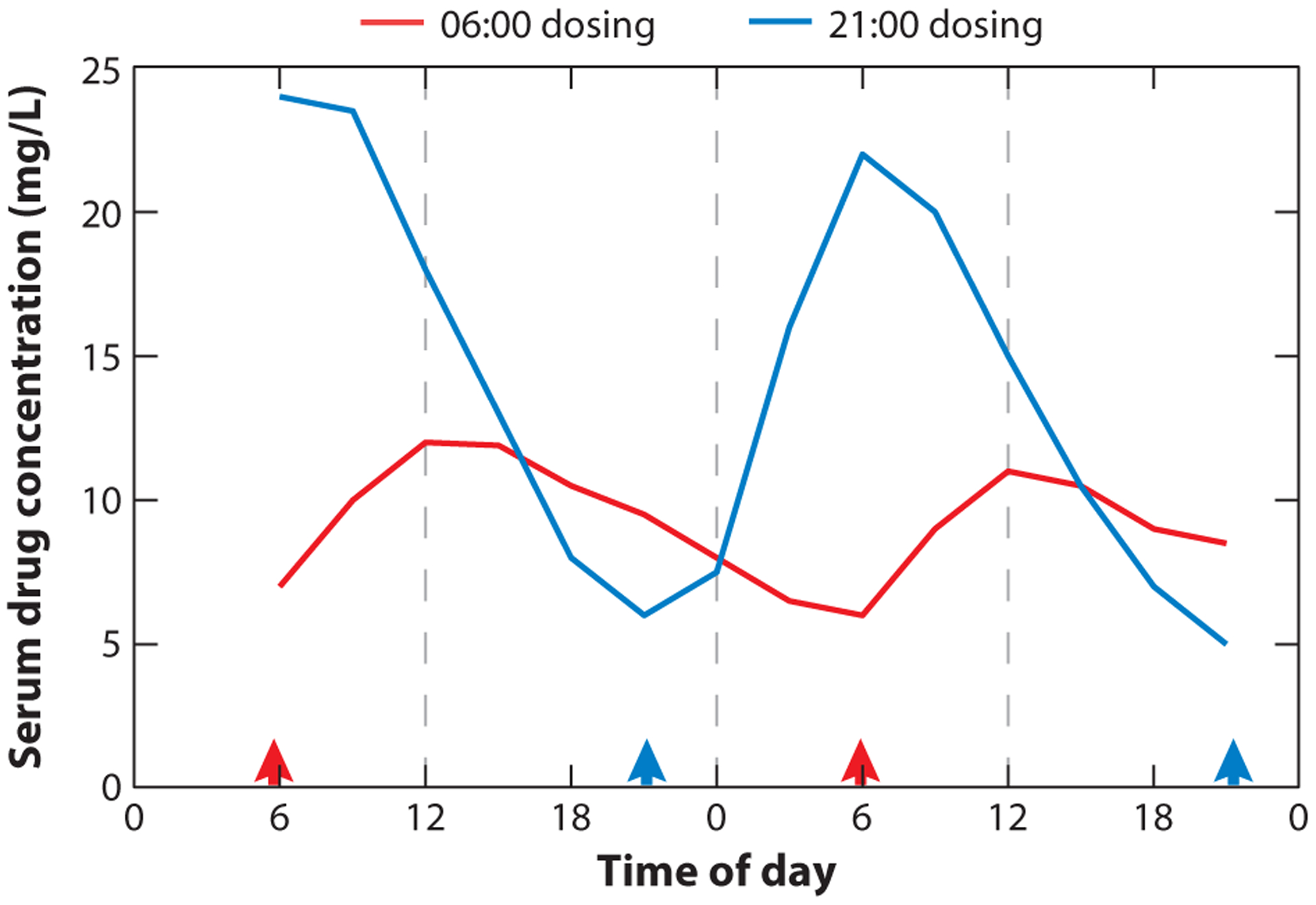
Time-of-day-dependent variations in pharmacokinetics. Steady-state serum concentrations of theophylline from eight asthmatic children dosed with the drug either before breakfast (06:00, *red*) or before bedtime (21:00, *blue*), as indicated by arrowheads. Bedtime dosing shows elevated serum concentrations correlating with the natural rhythm of dyspnea. Figure adapted from Reference 48.

PK parameters associated with the absorption, distribution, metabolism, and excretion of drugs often depend on the time of day of administration (43). Whether oral or parenteral, absorption kinetics often depend on the physiological processes of administration, which in turn are regulated in a temporal manner. Key transporters are expressed in a way that makes their levels peak at specific times during the day (44); as a result, critical PK parameters such as *C*_max_, *τ*_max_, AUC, and Cl (which define the maximum drug concentration, the time needed to reach that concentration, the area under the curve of the drug, and the clearance rate, respectively) depend strongly on the time of day of administration (45). For example, a recent study of asthmatic children dosed with theophylline at either 06:00 or 21:00 showed twofold-higher serum levels of the drug when dosed at 21:00. This night dosing regimen also correlated with the natural rhythm of dyspnea, peaking during the worst symptoms (Figure 3).

In a similar vein, drug metabolism and excretion are regulated by enzymes that, in turn, are regulated by clock genes, making these drug-processing genes peak at specific times during the light/dark periods. However, the PD implications, especially adverse effects, of the time of drug delivery can become far more pronounced (46, 47). For example, acetaminophen, theophylline, and ampicillin show different PK in morning versus evening dosage (48). Acetaminophen exhibits clear time-dependent toxicity in mouse models arising from the rhythmic expression of cytochrome 2E1 in the liver, which elevates production of NAPQI (*N*-acetyl-*p*-benzoquinone imine) (49). The molecular basis of this behavior is beginning to be understood and is thought to be driven in part by clock-regulated transcription factors such as PARbZip (proline and acidic amino acid–rich basic leucine zipper) binding to D-box promoter elements of the constitutive androstane receptor, cytochrome 3A4, and multidrug resistance mutation 1 in the liver. Similarly, bile acid production and excretion oscillate because of the rhythmic expression of REV-ERBα and FXR, leading to diurnal patterns in ampicillin and flomoxef biliary clearance (48). Interestingly, recent research has demonstrated clock-independent cyclic accumulation of triglycerides in murine livers, leading to time-of-day-dependent fatty liver (50), which increases the risk of certain toxicological endpoints such as steatosis or cholestasis during specific periods of the day.

Interestingly, the complex interdependence of PK, PD, and disease mechanisms manifests strongly in the context of viral infections and vaccination (51–53), where the innate antibody response couples with the ability of the virus to interfere with homeostasis. Note that, despite its complexity, the PK of a drug is better characterized than the PD, as the former usually involves somewhat well-understood physiological function and the latter often involves complex and generally unknown mechanisms of actions for both the drug and the disease.

The strong dependence of PK on circadian timing, so-called chronopharmacokinetics, has led to the realization that timing is a critical factor in optimal delivery of a drug in order to match appropriate PK parameters in a wide range of treatments, including anticancer, antiviral, and antibiotic agents; cardiovascular drugs and glucocorticoids; autonomic and respiratory agents; antiepileptic and thrombolytic drugs; and immunosuppressive agents (54). A very interesting complication in PK/PD dynamics arises from the fact that the nature of circadian variability and the PK/PD circadian dependence require the paradigm of a physiological baseline to be reevaluated (55). In other words, homeostatic time-of-day circadian variations may mask temporal dynamics of the drug and/or dose–response effects (56). The convolution of the abovementioned ideas and observations has led to novel ways to take advantage, or minimize the impact, of the interplay between xenobiotic metabolism and circadian rhythms (57).

**Figure 4:**
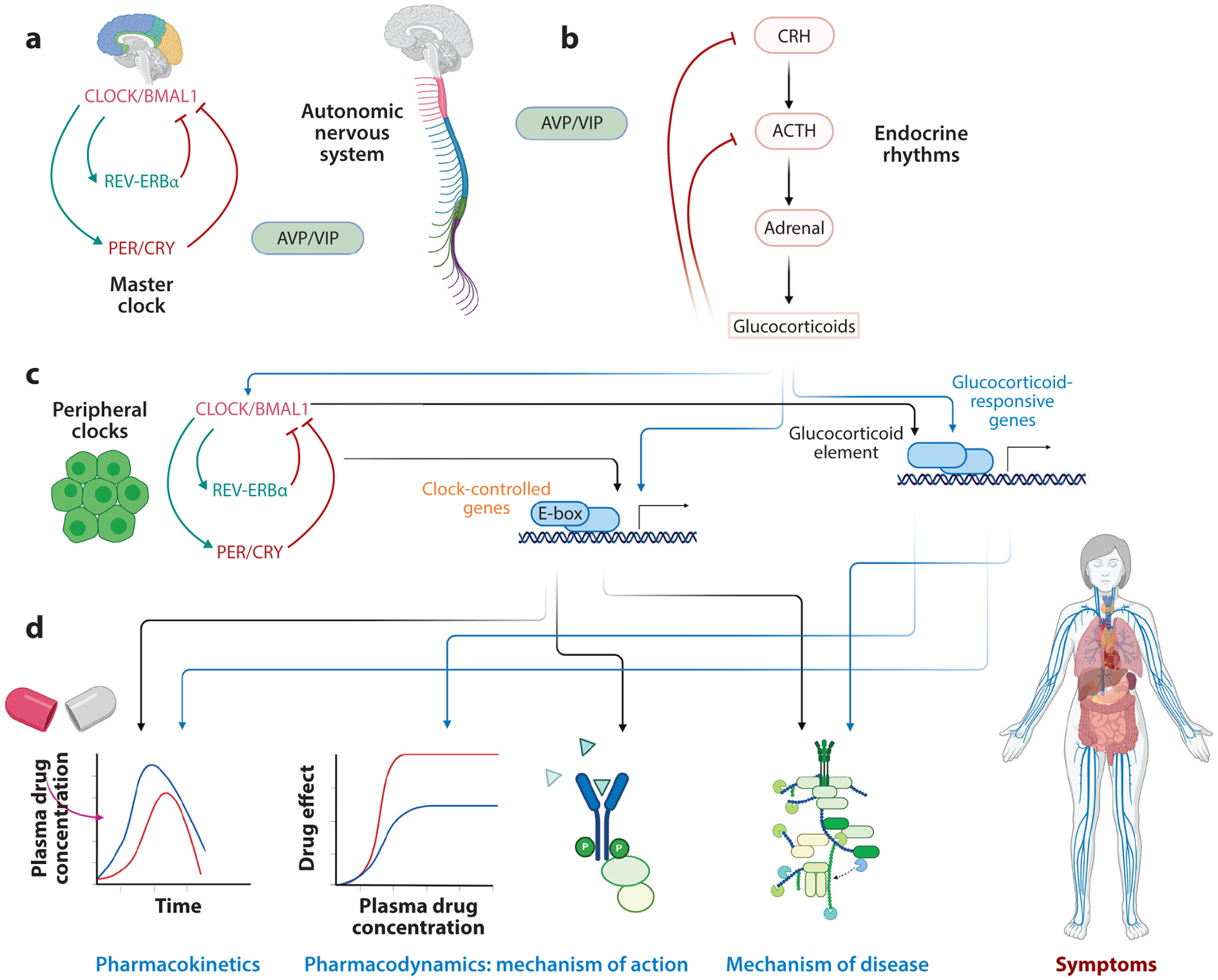
(*a*) The master clock, located in the suprachiasmatic nucleus, receives photic signals and is entrained by environmental cues. (*b*) It produces signals that subsequently entrain the autonomic and endocrine systems. These systems, in turn, produce circadian signals that entrain clocks in the periphery. (*c*) The peripheral clocks, along with endocrine hormones (glucocorticoids), induce downstream rhythms (*d*) by regulating the expression of several genes that are involved in the pharmacokinetics and pharmacodynamics of the drug, as well as in the mechanism of action of the drug or disease. The convolution of these rhythms regulates the action of the drug on the disease symptoms. Because a human is an open system, interactions with the environment are critical for enhancing or diminishing these rhythms, with possible adverse effects. Abbreviations: ACTH, adrenocorticotropic hormone; AVP, arginine vasopressin; CRH, corticotropin-releasing hormone; VIP, vasoactive intestinal polypeptide. Figure adapted from images created with BioRender.com.

However, even though substantial effort has been invested in deciphering the circadian dependence of PK- and PD-related processes, investigators have realized that the disease mechanisms also exhibit strong time-of-day dependencies, which manifest in the way symptoms are observed throughout the day (58), since immune function can also be coordinated in a circadian manner (Figure 4). One of the most comprehensive studies demonstrating this dependency was conducted by Montaigne et al. (59). While studying the daytime variation of perioperative myocardial injury in cardiac surgery, they confirmed that “the incidence of major adverse cardiac events was lower in the surgery group than in the morning group” (59, p. 59). This study also confirmed that the propensity for sustaining an injury differed between the active day period and the evening rest period, with the likely culprit being the myocardial clock. It is therefore important to appreciate that therapeutics act on targets related to a mechanism associated with driving a disrupted phenotype, relative to homeostasis. Thus, the dynamics of disease manifestation is crucial for understanding the complexities of the therapeutic process.

Multiple studies have attempted to map the circadian nature of symptoms, beginning with one of the most fundamental sensations, and likely one of the first and most important therapeutic priorities: pain. Pain is a symptom that is crucial for clinical practice, as it underlies myriad disease states (60). The circadian dependence of pain has been demonstrated in animal models showing that the pain threshold is highest during the end of the resting period and lowest at the end of the active period. However, the picture in humans is somewhat murkier (60). Subgroups of diseases and patients exhibit more consistent behaviors, with rheumatoid arthritis patients reporting greatest pain in the morning at the beginning of active period (61) and osteoarthritis patients reporting more pain at the start of the evening rest period (62). Regardless, pain sensation is thought to be related to endogenous opioid peptide levels, which are known to be higher during the morning period in humans (63, 64); this likely has implications for treatment (65).

Arterial blood pressure is another physiological measurement of great relevance for overall health due to its close relation to organ damage and, of course, cardiovascular events. The use of chronotherapy has been suggested for the subset of hypertensive patients with elevated heart rate (66). Blood pressure is affected by many external factors, ranging from ambient temperature and physical activity to emotional state and meal composition, as well as by intrinsic factors, such as genetics, ethnicity, age, and sex. However, the blood pressure time course during the day is driven by the circadian clock due to entrainment by the SCN and oscillating hormones such as cortisol (67). This circadian dependence is abolished when the master clock is damaged. Regardless of gender or age differences, it is generally expected that in healthy individuals blood pressure declines to its lowest levels during rest and peaks during the start of the active period. Interestingly, this observation coincides with the well-established increased occurrence of acute myocardial infarction and sudden cardiac death during the morning hours (68) as well as with a peak in the occurrence of ventricular premature beats between 06:00 and 12:00 (69). The list of circadian manifestations of symptomatology is rather long, with exacerbation of inflammation and subsequent increase in rhinitis and asthma symptoms during the early morning hours (70) in line with increased occurrence of rheumatoid arthritis symptoms (71).

Therefore, given the strong circadian dependence of not only the drug’s bioavailability and efficacy but also the disease mechanism, it is unsurprising that a substantial body of research has attempted to optimize drug delivery to match either (or both) of the time-of-day dependencies. One of the earliest attempts to optimize the temporal delivery of a drug so as to minimize its potential adverse effects focused on the administration of glucocorticoids such as prednisone and methylprednisolone (72). Side effects in these studies were substantially reduced without compromising efficacy. Several other studies also attempted to match the drug’s PK to time-of-day-dependent symptoms. Myocardial infarction, ischemic events, and sudden cardiac death all exhibit strong temporal segregation, with concentrations much higher toward the transition between the inactive night and active day periods (73). In one study (74), an optimal synergy among autonomic rhythms, guiding cardiovascular dynamics, and PK of aspirin was achieved when the drug was administered during the early morning hours to provide appropriate platelet inhibition, which likely led to a reduction in cardiovascular events. Similarly, in another study (75), several antihypertensive mechanisms of actions had a strong time-of-day dependence, whereas the efficacy and dose–response curves of individual drugs (e.g., valsartan, doxazosin) depended on the circadian time of drug administration. Cancer therapeutics is another area that has received increased attention, not only because of the likelihood of maximizing interference with tumor growth mechanisms but also because of the opportunity to minimize adverse effects (46, 47, 76–80).

The circadian dependence of critical nodes in a disease mechanism also points to circadian PD dependencies. Gout attacks, for example, are more pronounced during the rest (night/dark) period and are likely related to uric acid metabolism (81). Interestingly, genes such as xanthine dehydrogenase (*Xdh*) and urate oxidase (*Uox*), which are critical for clearing uric acid, have also been recognized as pharmacologic targets (82). Hyperuricemia drives acute gout flares. Recent studies have revealed that the risk of acute gout flares during the night and early morning hours is 2.4 times higher than in the daytime (81). Several factors, including body temperature, sleep, and cortisol levels, may be responsible, but a fundamental challenge remains: Gout flares have circadian rhythms. A better understanding of these time-of-day dependencies will significantly improve our understanding of the disease itself and will have implications for antigout prophylactic and treatment approaches.

## THE CIRCADIAN BASIS OF CHRONOPHARMACOLOGY

The goal of our discussion is not to present a comprehensive summary of circadian biology and circadian rhythms; we provide several significant references on the subject for further exploration. Instead, we focus on some of the exciting opportunities that have emerged since the tight connections between circadian rhythms and disease were discovered, especially as they relate to lifestyle-induced circadian disruption, overall health, and chronic diseases (83).

**Figure 5:**
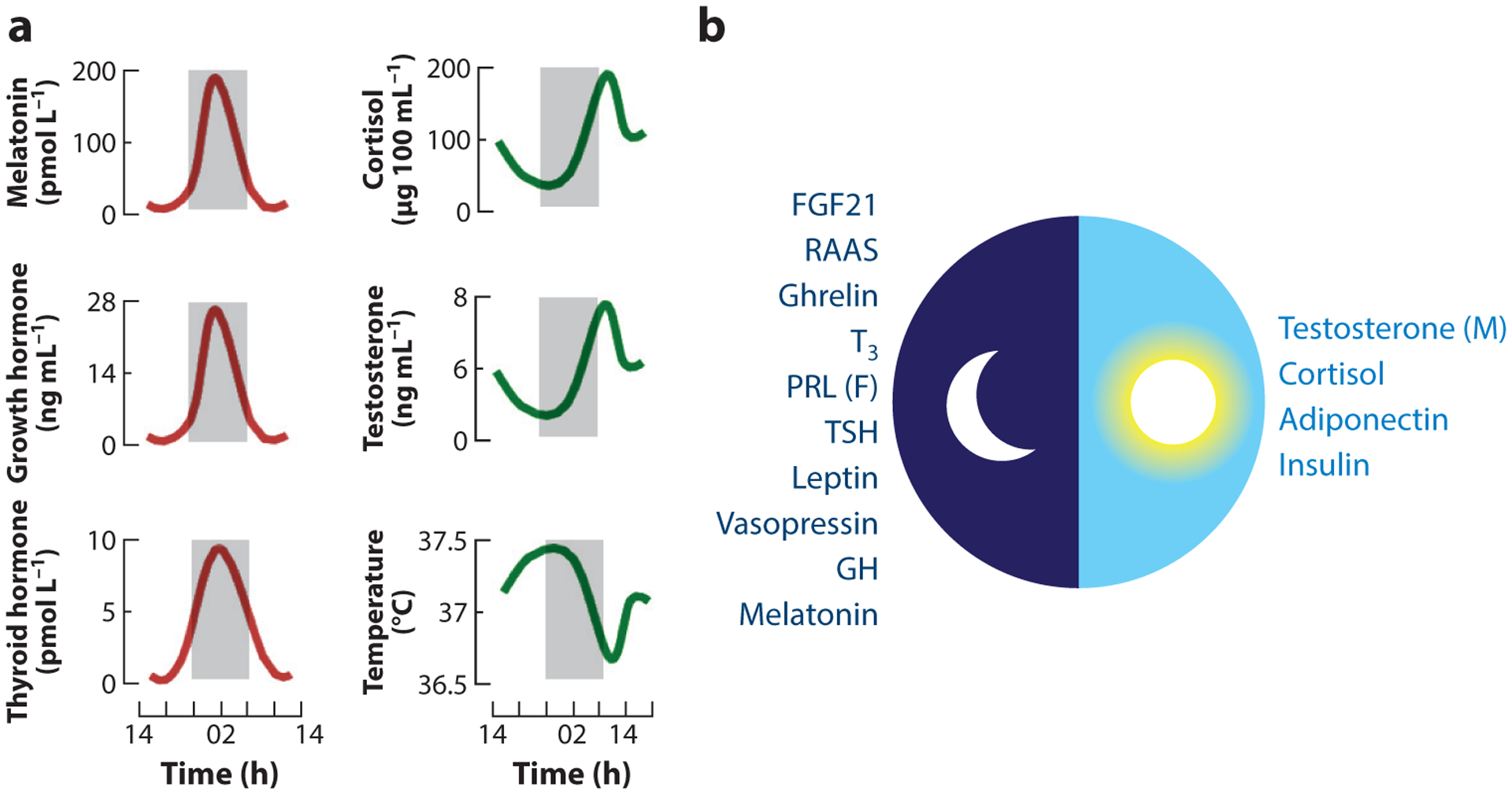
(*a*) Biological rhythms of certain physiological functions and hormone serum levels. (*b*) Peak hormone levels during the day/night cycle. Abbreviations: F, females; FGF, fibroblast growth factor; GH, growth hormone; M, males; PRL, prolactin; RAAS, renin–angiotensin–aldosterone system; TSH, thyroid-stimulating hormone. Panel *a* adapted from Reference 157. Panel *b* adapted from Reference 158.

In order to improve our understanding of the importance of circadian rhythms in human physiology and, in particular, the interactions between health and therapeutics, it is important to realize the tight connection between physiological mechanisms and biological rhythms. Figure 4 presents a simplified version of this mapping. The master clock constitutes, as mentioned above, an entrainable oscillator. This means that while SCN cells possess regulatory structures that can produce sustained oscillations (Figure 1), their phase and period can be set by external signals, such as light/dark patterns or feeding schedule. The master clock conveys these rhythmic signals to the periphery through autonomic and neuroendocrine signals (84). The latter are particularly important because they determine the rhythmic production of endocrine mediators (Figure 5). Interestingly, the endocrine system itself is a self-sustained oscillator due to the activity of negative feedback loops (85). Figure 4 depicts a simplified model of the hypothalamic–pituitary–adrenal (HPA) axis. An example of a negative feedback loop in this axis is control of thyroid hormone secretion. The hypothalamus secretes thyroid-releasing hormone (TRH), stimulating the pituitary gland to secrete thyrotropin, the thyroid-stimulating hormone (TSH). TSH stimulates the secretion of thyroid hormones from the epithelial cells of the thyroid gland, which signals neurons in the hypothalamus to stop secreted TRH, resulting in natural oscillations.

SCN signals set the phase and period of endocrine hormone secretion. For example, cortisol increases from low levels during the night period to peak during the early morning hours, concomitantly driving an increase in systolic blood pressure; in contrast, levels of melatonin, growth hormone, and TSH peak during the middle of the night, driving the sleep cycle, metabolic changes, and tissue growth (Figure 5). This cycle is driven by two SCN-derived neurotransmitters, arginine vasopressin and vasoactive intestinal peptide, that negatively regulate corticotropin-releasing hormone, which controls cortisol secretion. The secretion of cortisol entrains the hormone to exhibit a circadian pattern of activity (86, 87), resulting in a precise peak of cortisol/corticosterone at the beginning of the active phase. In turn, glucocorticoids influence the function of virtually all organs and tissues and are critical for most of the physiological activities that coordinate and propagate physiological rhythms, and phase relationships, among downstream processes across the host. When rhythms become irregular in the SCN, the input to the hypothalamus becomes distorted, resulting in cortisol rhythm alterations in phase, amplitude, or period that in turn are linked to numerous pathologies (88, 89). Note, however, that regulation of the HPA axis is more complex (90).

All peripheral tissues also contain their own timekeeping mechanisms, which need to be entrained to the proper phase relations by systemic signals, especially endocrine factors. Finally, clock-controlled and/or glucocorticoid-responsive genes regulate myriad functions at the tissue and organ levels. Therefore, this cascading and distributed structure conveys and coordinates rhythmic information that eventually affect processes driving the PK and PD of the drug, the mechanism of action of the drug, and innate physiological mechanisms, deregulation of which leads to disease. Disease may give rise to mediators that further disrupt the homeostatic rhythmic patterns, inducing a vicious cycle propagating circadian disruption (Figure 4) (91). The renewed interest in circadian disruption (92) stems from the realization that it is associated with longer-term detrimental health effects (93, 94) and, in particular, with the accumulation of allostatic load (95, 96).

Having established the importance of circadian rhythms in general, as well as their role in PK/PD and disease, we now articulate a series of questions that are expected to play a dominant role in our effort to not only better understand but also capitalize on the synergies between circadian rhythms and pharmacology.

### Circadian Baseline

Traditionally, PK/PD studies focused on assessing effects of dose and route. For good reasons, a baseline needs to be established so that rational comparisons across conditions can be made. However, as it is becoming increasingly well appreciated that several physiological functions associated with drug absorption, distribution, metabolism, and excretion are regulated by the circadian clock, both study designs and the baseline should be modified appropriately (45). For example, the expression of cytochrome P450 (CYP450) enzymes that control drug metabolism in the liver is circadian, controlled in part by the oscillations of PPARα, CCAAT enhancer–binding proteins α and β, hepatocyte nuclear factor 1α, and RORα/γ, which are directly connected to the cell-autonomous clock (Figure 1). Drug absorption and excretion are similarly affected by circadian rhythms, with gastric activity peaking at night and blood flow, bile excretion, and renal activity peaking during the active period.

Circadian fluctuations need to be taken into account more systematically as PK models are developed, which will lead to dose analyses for clinical studies (97). To this end, investigators will need to revisit current experimental, clinical, and modeling approaches in order to best account for circadian PK/PD variation and to optimize treatment (55, 98). Also, we now know that disease condition can alter baseline characteristics. Studies in critical care patients whose circadian rhythms are effectively abolished (99) have shown that the circadian PK/PD behavior of pharmaceuticals tends to diminish (100).

### Age, Gender, and Ethnicity

Differences in genetics, including gender and ethnic background, play an important role in PK/PD (101), as does age (102). The physiology of elderly persons and toddlers often differs from that of the general population because of behavioral, dietary, and hormonal differences, leading to varying circadian rhythm patterns (103). Age is expected to become an extremely important confounder in the near future, as the elderly population is expected to double by 2050 (104). Physiological parameters associated with aging include loss of amplitude of oscillation and circadian phase delay (103). Elderly patients show diminished melatonin and testosterone production, leading to sleep disorders and metabolic changes; in contrast, cortisol levels increase with age, disrupting circadian effects studied in the general population. While the underlying biological mechanisms are still being investigated, the implications for PK/PD are clear, as these mechanisms are involved not only in maintaining an already frail homeostasis (105) but also in pharmacological activity.

Furthermore, a strong link between circadian rhythms and gender has been established, especially in relation to disease (106) and race (107). Therefore, circadian dependencies extend beyond broader biological structures, and dominant subcategories are emerging. Recent studies have begun to shed light on issues related to ethnicity by assessing the characteristics of the endogenous circadian clock of individuals of European versus African heritage. Interestingly, these studies identified fundamental differences not only in circadian characteristics but also, more importantly, in the ability of the clocks to adapt depending on heritage (108–110). Further studies have demonstrated the potential impact of gender and race on circadian misalignment, such as the disruption of signals to the SCN by sleep perturbations, which may have ancestry-dependent implications (111, 112). However, note that despite observed differences in the context of circadian rhythms, early PK studies have not established statistically significant effects of age and ethnicity on PK (113–115). Integrated studies have begun to articulate the powerful combination of in vivo experiments and complex PK/PD modeling for assessing the impact of combinations of factors such as gender (116) as well as lifestyle and diet (117).

### Impact of Stress

Stress, whether physical or psychological, interferes with the regulation of circadian rhythms (118, 119) mainly because of its effect on the stress hormone cortisol. Cortisol production affects blood flow and vascular function, thus influencing drug distribution; drug metabolism, by modulating CYP450 activity; and drug excretion, due its effect on bile production. Cortisol can also affect a drug’s mechanism of action and other PD parameters as a result of its critical role in metabolic function (120, 121). These observations raise the possibility of enriching the paradigm to account for stress. That should come as no surprise, since stress is a major contributor to overall homeostasis and homeostatic mechanisms.

### Replicating Circadian Rhythms

**Figure 6:**
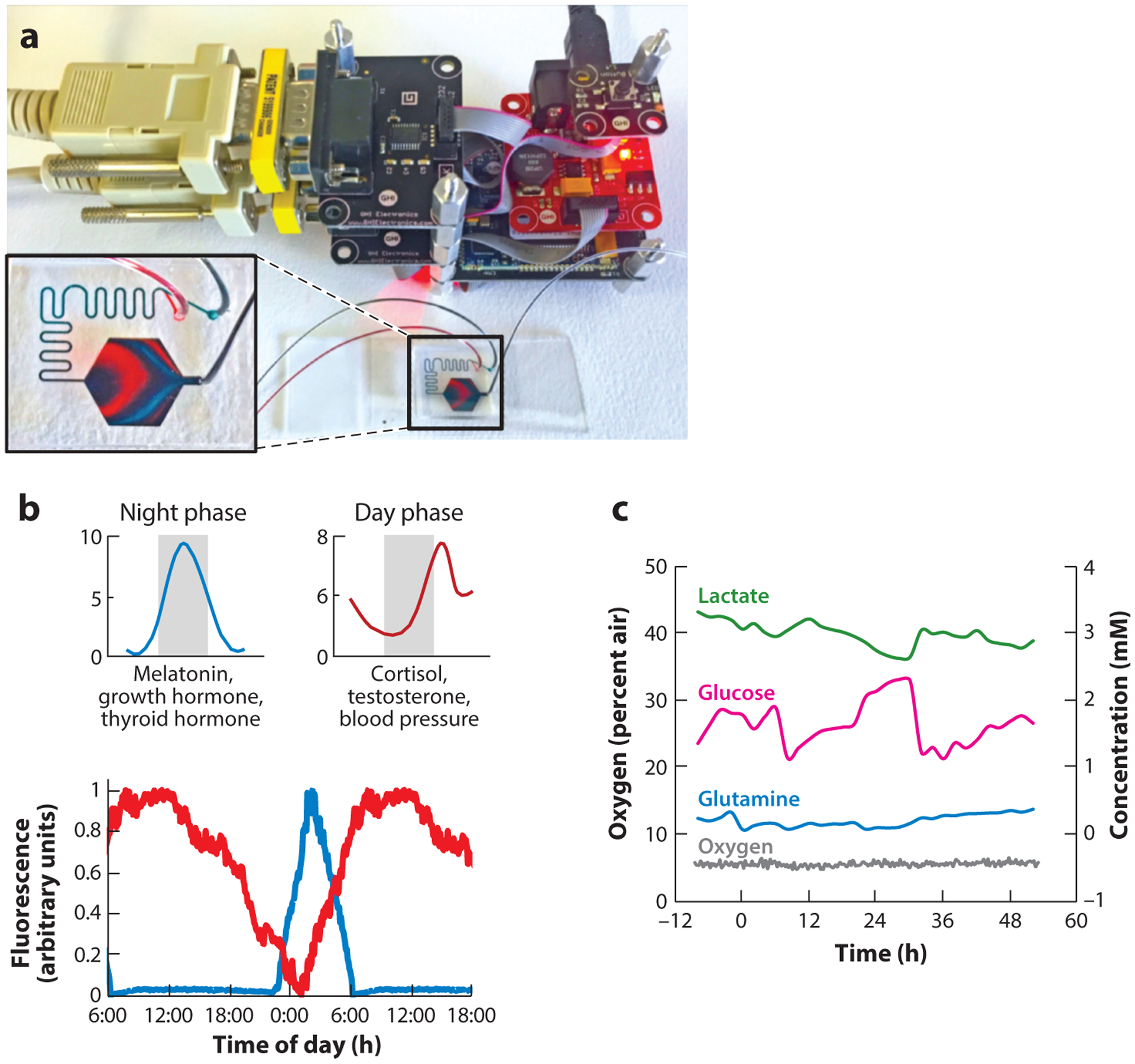
(*a*) Computer-controlled microfluidics mimicking the complex dynamics of hormonal oscillations using a simple microfluidic mixer. The night phase is labeled red; the day phase is labeled blue. (*b*) (*Top*) Schematic and (*bottom*) experimental validation of hormone patterns in a liver-on-a-chip microdevice connected to a mixer. (*c*) Real-time measurements of lactate production, glucose consumption, glutamine uptake, and oxygen in HepG2/C3A liver spheroids on a chip using embedded sensors (130). The spheroids demonstrate metabolic oscillations with a 24-h phase. Figure adapted courtesy of the Nahmias lab and Tissue Dynamics.

The above discussion clearly demonstrates that circadian regulation is a multifactorial, multilayered process with many moving parts. However, drugs often target or mediate the function of certain critical aspects of these components. One such aspect that has received significant attention comprises the endocrine system and cortisol (122), as synthetic analogs of glucocorticoids are routinely used for several acute and chronic conditions (123). Glucocorticoid administration in inflammatory conditions has focused primarily on targeting symptom onset. However, limited studies have shown that the circadian-dependent effects on circulating cortisol levels can become very pronounced depending on the time of day synthetic glucocorticoids are administered (71). Such observations have raised the possibility of timing drug administration not solely with the purpose of modulating symptoms but also to help restore homeostatic rhythms of endogenous mediators, which may be directly or indirectly affected by the drug (124). Large-scale human studies have indicated that if glucocorticoid administration is optimized to enhance the synchronization of the drug, leading to enhanced peripheral circadian rhythms, it could improve the drug’s efficacy, especially in conditions requiring long-term treatment (125). Melatonin is dosed at night, as close as possible to its circadian peak, in the treatment of sleep disorders, and circadian dosing of thyroid medication is currently being studied by clinicians as well as patient groups (126). In this respect, detailed modeling studies and novel microfluidics devices (Figure 6) can provide significant insight in order to optimize different treatment regimens that derive new benefits from existing medications (see the sidebar titled Circadian Rhythms on a Chip) (127, 128).

### Drug the Clock

The realization that the circadian clock, and its dysregulation, plays a central role in several pathologies (94, 131) has given rise to several possible ways to target the characteristics of the clock itself (132, 133) regardless of, to some extent, the details of the pathology. As a result, it has become possible to study and elucidate the actions of a wide family of small molecules that directly affect components of the circadian clock (132–138). Several nonpharmacologic interventions, mostly nutritional supplements, have emerged as potential regulators of nuclear receptors that influence the cell-autonomous clock (Figure 1) (139).

### Personalized Pharmacokinetics and Pharmacodynamics

A major insight obtained from in-depth analysis of the role of circadian rhythms is that the dynamics of the presentation of the drug needs to be incorporated into the analysis. In other words, it does not suffice to describe who the players are; it is equally important to appreciate and understand how they interact. Systems chronotherapeutics is therefore thought to play a substantial role (140) as we attempt to integrate numerous components across a multitude of tissues and organs. Advanced computational methods would greatly aid attempts to rationalize complex clinical studies and to develop appropriate PK/PD mathematical models that describe variations of a wide range of circadian characteristics as we develop a better understanding of the implications of circadian rhythms at a personalized level (141–143). Early ideas have provided support to approaches that would tailor drug administration on the basis of a patient’s chronotype, that is, an individual’s behavioral manifestation of circadian rhythms (45). In many ways, this is a physiologically focused variant of personalized medicine, looking at patients’ behavioral patterns in addition to their genetics.

**CIRCADIAN RHYTHMS ON A CHIP**

Circadian rhythms are commonly studied using small animals. However, differences in physiology, metabolism, and genetics as well as an inverted day/night cycle make it difficult to translate findings to human patients. While human cells can be synchronized in vitro using serum starvation of high-dose cortisol, synchronization is lost within 48 to 72 h. In addition, the hormonal response induced by cortisol is a small part of the complex rhythms of temperature and hormones. Recently, Cyr et al. (129) developed the MES-μF (Missing Endocrine System MicroFormulator), which can create time-resolved combinations of hormones in multiwell plates. This technology has been licensed by CN Bio Innovations into its PhysioMimix^™^ instrument. The Nahmias lab has developed a similar platform based on microfluidic mixing of day and night hormones to create stable oscillations (Figure 6). The technology has been licensed by Tissue Dynamics into its DynamiX^™^ instrument.

### Chronotherapeutic Drug Delivery Systems

This brings us to what may be the most important question: Do we need specialized drug delivery systems to implement chronotherapy and truly maximize its impact? Although the ideas have been outlined and the benefits articulated, in order to develop appropriate dosing schedules that match circadian rhythms, patient adherence is paramount (144), necessitating the development of engineered constructs that exhibit periodic release characteristics.

In general, chronotherapeutic drug delivery systems tend to favor delayed- or controlled-release formulations; however, advanced systems need to incorporate a real-time triggering biomarker to activate on-demand release (145). Examples of such constructs include chronoprogrammable electronic pumps that control drug infusion (146). Other alternatives, in principle, involve the selection of hydrogels with an appropriate response, such as swelling, to the environmental rhythms of the surrounding fluids (147).

Chronotherapeutics, that is, timing bioavailable concentrations to match disease symptoms, has been seriously considered for several treatments, including anti-inflammatory therapies using nonsteroidal anti-inflammatory drugs to match the drug with the expected cytokine peak; asthma, due to variance in immune function; cardiovascular disease, due to daily variation in blood pressure; dyslipidemia, due to the circadian nature of cholesterol metabolism; and cancers, due to the circadian pattern of the adverse effects of chemotherapy (148). However, most current paradigms still rely on controlled release rather than taking advantage of internal circadian changes in drug absorption or metabolism. For drug delivery schemes that aim to take advantage of circadian patterns, we need to (*a*) understand the circadian nature of the PK/PD and disease/drug mechanisms so as to optimize timing of therapy to maximize efficacy, (*b*) identify novel pharmacologic compounds that target the circadian mechanism, and (*c*) identify novel biomarkers that enable us to truly characterize the circadian state of the system (149).

### Novel Biomarkers

Circadian rhythms play a role in the regulation of many physiological functions, and numerous biomarkers exhibit strong circadian patterns. However, in order to advance the field it will be important to accurately scope and characterize time-of-day-dependent changes in myriad physiological functions. Although markers have been developed to assess deviations from homeostasis, enabling the quantification of symptoms and the impact of therapy, the ability to characterize patients’ circadian status is currently unavailable. Any discussion of circadian patterns and their disturbance assumes the concurrent existence of two groups (sham and treated), and all discussion is presented through a comparison of the two groups’ complete 24-h waveforms of invasive measurements of relatively high temporal sampling resolution. Therefore, the concept of a biomarker is still lacking in the context of circadian rhythms (i.e., Are there unique markers that would characterize either the entire system or subsystems of interest?). Establishing such markers will be critical to strengthen the functional relations between chronotype and circadian effects on the drug and disease (150).

The human blood transcriptome has been the focus of many studies (150, 151), given that blood draws are among the least invasive and most straightforward methods for sample collection. However, an interesting question remains as to which component of a blood draw would be the best representative of the circadian timing. A major challenge of most current approaches is that they rely on collecting temporal data over the entire circadian cycle, which substantially increases the sampling burden and makes quick decisions difficult. Therefore, recent methods have attempted to develop bioinformatics tools that can assess circadian timing from a single sample (152). Along this line, researchers (153) have proposed the development of markers that detect the actual circadian time, a highly relevant issue, since this measure is the most important. Finally, very exciting recent results (154) demonstrate that the skin offers accurate information regarding the circadian status of the system and can represent an excellent avenue for a minimally invasive system to assess the circadian state of the system and, possibly, for the development of drug delivery mechanisms that can use this information to deliver on-demand drugs (much like implantable insulin infusion devices.)

## CONCLUSIONS

Recently, there has been renewed interest in the circadian system. Biological rhythms are not new. Since the seminal research by the Hall and Rosbash group (155) and the Young group (156), who were the first to determine the cellular mechanisms underlying circadian rhythms, a large body of work has explored the evolutionary origins and downstream implications of circadian rhythms. However, we have recently started to appreciate the broader role circadian rhythms play in maintaining health, coordinating physiological functions, and contributing to disease. We also realize that circadian rhythms manifest as the emergence of a complex equilibrium that can easily be disrupted by several behavioral factors that appear to be common in everyday life. Furthermore, various diseases, particularly chronic ones, tend to disrupt this circadian coordination. The presence of homeostatic rhythms, and the fact that PK and PD also possess their own rhythms, creates appealing opportunities in terms of matching pharmacologic interventions to innate rhythms in order to maximize efficacy and minimize adverse effects. The purpose of our review has been to set the stage in terms of the relations among circadian rhythms, homeostatic mechanisms, and a drug’s PK/PD and then to discuss various opportunities and their associated challenges that emerge from our attempt to effectively capitalize on circadian rhythms. Chronopharmacology is part of the future of personalized medicine. However, there remain several key questions to be addressed and multiple obstacles to be overcome.
